# Supplementary material for: Dynalign II: common secondary structure prediction for RNA homologs with domain insertions
Source: Nucleic Acids Res. 2014 Nov 21;42(22):13939–48. doi: 10.1093/nar/gku1172 (PMC4267632; doi:10.1093/nar/gku1172)
Supplement: SUPPLEMENTARY DATA [file supp_gku1172_nar-02021-z-2014-File012.zip › manual/javadoc/RNAstructure_java_drawing/source/dialogs/StructureDialog.html]

StructureDialog


JavaScript is disabled on your browser.


- Overview
- Package
- Class
- Tree
- Index
- Help

- Prev Class
- Next Class

- Frames
- No Frames

- All Classes

- Summary:
- Nested |
- Field |
- Constr |
- Method

- Detail:
- Field |
- Constr |
- Method


RNAstructure\_java\_drawing.source.dialogs

## Class StructureDialog

- java.lang.Object
- - java.awt.Component
  - - java.awt.Container
    - - java.awt.Window
      - - java.awt.Dialog
        - - javax.swing.JDialog
          - - RNAstructure\_java\_drawing.source.dialogs.ImageDialog
            - - RNAstructure\_java\_drawing.source.dialogs.StructureDialog

- All Implemented Interfaces:
  :   ImageObserver, MenuContainer, Printable, Serializable, Accessible, RootPaneContainer, WindowConstants

  ---

    

  ```
  public class StructureDialog
  extends ImageDialog
  ```

  A dialog that holds structure images.

  See Also:
  :   Serialized Form

- - ### Nested Class Summary

    - ### Nested classes/interfaces inherited from class javax.swing.JDialog

      `JDialog.AccessibleJDialog`
    - ### Nested classes/interfaces inherited from class java.awt.Dialog

      `Dialog.AccessibleAWTDialog, Dialog.ModalExclusionType, Dialog.ModalityType`
    - ### Nested classes/interfaces inherited from class java.awt.Window

      `Window.AccessibleAWTWindow, Window.Type`
    - ### Nested classes/interfaces inherited from class java.awt.Container

      `Container.AccessibleAWTContainer`
    - ### Nested classes/interfaces inherited from class java.awt.Component

      `Component.AccessibleAWTComponent, Component.BaselineResizeBehavior, Component.BltBufferStrategy, Component.FlipBufferStrategy`
  - ### Field Summary

    Fields

    | Modifier and Type | Field and Description |
    | --- | --- |
    | `private StructureBackend` | `backend` The back end object that reads data for structures. |
    | `static boolean` | `CIRCLED_NUCS` Boolean that tells whether a structure's nucleotides are circled. |
    | `static boolean` | `CLOCKWISE_STRUCTURE` Boolean that tells whether a structure is rendered clockwise. |
    | `private String[]` | `data` The array of data for a particular structure. |
    | `private boolean` | `hasPairs` Whether the current structure has pairs. |
    | `private int` | `number` The structure number viewed on this panel. |
    | `private String[]` | `regex` The array of regexes used to pull data about a structure. |
    | `private static long` | `serialVersionUID` |

    - ### Fields inherited from class RNAstructure\_java\_drawing.source.dialogs.ImageDialog

      `error, file, mainPanel, maxBoundX, maxBoundY, preferredDialogWidth, scale`
    - ### Fields inherited from class javax.swing.JDialog

      `accessibleContext, rootPane, rootPaneCheckingEnabled`
    - ### Fields inherited from class java.awt.Dialog

      `DEFAULT_MODALITY_TYPE`
    - ### Fields inherited from class java.awt.Component

      `BOTTOM_ALIGNMENT, CENTER_ALIGNMENT, LEFT_ALIGNMENT, RIGHT_ALIGNMENT, TOP_ALIGNMENT`
    - ### Fields inherited from interface java.awt.print.Printable

      `NO_SUCH_PAGE, PAGE_EXISTS`
    - ### Fields inherited from interface javax.swing.WindowConstants

      `DISPOSE_ON_CLOSE, DO_NOTHING_ON_CLOSE, EXIT_ON_CLOSE, HIDE_ON_CLOSE`
    - ### Fields inherited from interface java.awt.image.ImageObserver

      `ABORT, ALLBITS, ERROR, FRAMEBITS, HEIGHT, PROPERTIES, SOMEBITS, WIDTH`
  - ### Constructor Summary

    Constructors

    Constructor and Description

    `StructureDialog(String file)`

    Constructor.
  - ### Method Summary

    Methods

    | Modifier and Type | Method and Description |
    | --- | --- |
    | `void` | `clearAnnotation()` Clear annotation from this dialog. |
    | `protected void` | `createDrawnImage(Graphics2D g2)` Create the image drawn on this panel. |
    | `void` | `flip()` Flip the structure image horizontally. |
    | `protected void` | `readImageData()` Read image data from a particular file. |
    | `void` | `setAnnotation(String file)` Set the annotation for this structure drawing. |
    | `void` | `setStructureNumber(int number)` Set the structure number. |
    | `void` | `switchCircled()` Switch circled nucleotides on or off. |
    | `void` | `writeDotBracketFile(String outFile)` Write a dot bracket file of all structures on this dialog. |
    | `void` | `writeHelixFile(String outFile)` Write a helix file from the current structure on this dialog. |
    | `void` | `writePostscriptFile(String outFile)` Write a Postscript file from the current structure on this dialog. |
    | `void` | `writeSVGFile(String outFile)` Write an SVG file from the current structure on this dialog. |

    - ### Methods inherited from class RNAstructure\_java\_drawing.source.dialogs.ImageDialog

      `buildLegend, createDefaultMenuBar, getFile, getScale, isError, print, printPanel, refresh, setScale, setTopCaption, viewDialog, viewZoomDialog, zoomImage`
    - ### Methods inherited from class javax.swing.JDialog

      `addImpl, createRootPane, dialogInit, getAccessibleContext, getContentPane, getDefaultCloseOperation, getGlassPane, getGraphics, getJMenuBar, getLayeredPane, getRootPane, getTransferHandler, isDefaultLookAndFeelDecorated, isRootPaneCheckingEnabled, paramString, processWindowEvent, remove, repaint, setContentPane, setDefaultCloseOperation, setDefaultLookAndFeelDecorated, setGlassPane, setJMenuBar, setLayeredPane, setLayout, setRootPane, setRootPaneCheckingEnabled, setTransferHandler, update`
    - ### Methods inherited from class java.awt.Dialog

      `addNotify, getModalityType, getTitle, hide, isModal, isResizable, isUndecorated, setBackground, setModal, setModalityType, setOpacity, setResizable, setShape, setTitle, setUndecorated, setVisible, show, toBack`
    - ### Methods inherited from class java.awt.Window

      `addPropertyChangeListener, addPropertyChangeListener, addWindowFocusListener, addWindowListener, addWindowStateListener, applyResourceBundle, applyResourceBundle, createBufferStrategy, createBufferStrategy, dispose, getBackground, getBufferStrategy, getFocusableWindowState, getFocusCycleRootAncestor, getFocusOwner, getFocusTraversalKeys, getIconImages, getInputContext, getListeners, getLocale, getModalExclusionType, getMostRecentFocusOwner, getOpacity, getOwnedWindows, getOwner, getOwnerlessWindows, getShape, getToolkit, getType, getWarningString, getWindowFocusListeners, getWindowListeners, getWindows, getWindowStateListeners, isActive, isAlwaysOnTop, isAlwaysOnTopSupported, isAutoRequestFocus, isFocusableWindow, isFocusCycleRoot, isFocused, isLocationByPlatform, isOpaque, isShowing, isValidateRoot, pack, paint, postEvent, processEvent, processWindowFocusEvent, processWindowStateEvent, removeNotify, removeWindowFocusListener, removeWindowListener, removeWindowStateListener, reshape, setAlwaysOnTop, setAutoRequestFocus, setBounds, setBounds, setCursor, setFocusableWindowState, setFocusCycleRoot, setIconImage, setIconImages, setLocation, setLocation, setLocationByPlatform, setLocationRelativeTo, setMinimumSize, setModalExclusionType, setSize, setSize, setType, toFront`
    - ### Methods inherited from class java.awt.Container

      `add, add, add, add, add, addContainerListener, applyComponentOrientation, areFocusTraversalKeysSet, countComponents, deliverEvent, doLayout, findComponentAt, findComponentAt, getAlignmentX, getAlignmentY, getComponent, getComponentAt, getComponentAt, getComponentCount, getComponents, getComponentZOrder, getContainerListeners, getFocusTraversalPolicy, getInsets, getLayout, getMaximumSize, getMinimumSize, getMousePosition, getPreferredSize, insets, invalidate, isAncestorOf, isFocusCycleRoot, isFocusTraversalPolicyProvider, isFocusTraversalPolicySet, layout, list, list, locate, minimumSize, paintComponents, preferredSize, print, printComponents, processContainerEvent, remove, removeAll, removeContainerListener, setComponentZOrder, setFocusTraversalKeys, setFocusTraversalPolicy, setFocusTraversalPolicyProvider, setFont, transferFocusDownCycle, validate, validateTree`
    - ### Methods inherited from class java.awt.Component

      `action, add, addComponentListener, addFocusListener, addHierarchyBoundsListener, addHierarchyListener, addInputMethodListener, addKeyListener, addMouseListener, addMouseMotionListener, addMouseWheelListener, bounds, checkImage, checkImage, coalesceEvents, contains, contains, createImage, createImage, createVolatileImage, createVolatileImage, disable, disableEvents, dispatchEvent, enable, enable, enableEvents, enableInputMethods, firePropertyChange, firePropertyChange, firePropertyChange, firePropertyChange, firePropertyChange, firePropertyChange, firePropertyChange, firePropertyChange, firePropertyChange, getBaseline, getBaselineResizeBehavior, getBounds, getBounds, getColorModel, getComponentListeners, getComponentOrientation, getCursor, getDropTarget, getFocusListeners, getFocusTraversalKeysEnabled, getFont, getFontMetrics, getForeground, getGraphicsConfiguration, getHeight, getHierarchyBoundsListeners, getHierarchyListeners, getIgnoreRepaint, getInputMethodListeners, getInputMethodRequests, getKeyListeners, getLocation, getLocation, getLocationOnScreen, getMouseListeners, getMouseMotionListeners, getMousePosition, getMouseWheelListeners, getName, getParent, getPeer, getPropertyChangeListeners, getPropertyChangeListeners, getSize, getSize, getTreeLock, getWidth, getX, getY, gotFocus, handleEvent, hasFocus, imageUpdate, inside, isBackgroundSet, isCursorSet, isDisplayable, isDoubleBuffered, isEnabled, isFocusable, isFocusOwner, isFocusTraversable, isFontSet, isForegroundSet, isLightweight, isMaximumSizeSet, isMinimumSizeSet, isPreferredSizeSet, isValid, isVisible, keyDown, keyUp, list, list, list, location, lostFocus, mouseDown, mouseDrag, mouseEnter, mouseExit, mouseMove, mouseUp, move, nextFocus, paintAll, prepareImage, prepareImage, printAll, processComponentEvent, processFocusEvent, processHierarchyBoundsEvent, processHierarchyEvent, processInputMethodEvent, processKeyEvent, processMouseEvent, processMouseMotionEvent, processMouseWheelEvent, remove, removeComponentListener, removeFocusListener, removeHierarchyBoundsListener, removeHierarchyListener, removeInputMethodListener, removeKeyListener, removeMouseListener, removeMouseMotionListener, removeMouseWheelListener, removePropertyChangeListener, removePropertyChangeListener, repaint, repaint, repaint, requestFocus, requestFocus, requestFocusInWindow, requestFocusInWindow, resize, resize, revalidate, setComponentOrientation, setDropTarget, setEnabled, setFocusable, setFocusTraversalKeysEnabled, setForeground, setIgnoreRepaint, setLocale, setMaximumSize, setName, setPreferredSize, show, size, toString, transferFocus, transferFocusBackward, transferFocusUpCycle`
    - ### Methods inherited from class java.lang.Object

      `clone, equals, finalize, getClass, hashCode, notify, notifyAll, wait, wait, wait`

- - ### Field Detail


    - #### backend

      ```
      private StructureBackend backend
      ```

      The back end object that reads data for structures.


    - #### CIRCLED\_NUCS

      ```
      public static boolean CIRCLED_NUCS
      ```

      Boolean that tells whether a structure's nucleotides are circled.


    - #### CLOCKWISE\_STRUCTURE

      ```
      public static boolean CLOCKWISE_STRUCTURE
      ```

      Boolean that tells whether a structure is rendered clockwise.


    - #### data

      ```
      private String[] data
      ```

      The array of data for a particular structure.


    - #### hasPairs

      ```
      private boolean hasPairs
      ```

      Whether the current structure has pairs.


    - #### number

      ```
      private int number
      ```

      The structure number viewed on this panel.


    - #### regex

      ```
      private String[] regex
      ```

      The array of regexes used to pull data about a structure.


    - #### serialVersionUID

      ```
      private static final long serialVersionUID
      ```

      See Also:
      :   Constant Field Values
  - ### Constructor Detail


    - #### StructureDialog

      ```
      public StructureDialog(String file)
      ```

      Constructor.

      Parameters:
      :   `file` - The structure file to draw.
  - ### Method Detail


    - #### clearAnnotation

      ```
      public void clearAnnotation()
      ```

      Clear annotation from this dialog.


    - #### createDrawnImage

      ```
      protected void createDrawnImage(Graphics2D g2)
      ```

      **Description copied from class: `ImageDialog`**

      Create the image drawn on this panel.

      **Specified by:**
      :   `createDrawnImage` in class `ImageDialog`

      Parameters:
      :   `g2` - The graphics context.


    - #### flip

      ```
      public void flip()
      ```

      Flip the structure image horizontally.


    - #### readImageData

      ```
      protected void readImageData()
      ```

      **Description copied from class: `ImageDialog`**

      Read image data from a particular file.

      **Specified by:**
      :   `readImageData` in class `ImageDialog`


    - #### setAnnotation

      ```
      public void setAnnotation(String file)
      ```

      Set the annotation for this structure drawing.

      Parameters:
      :   `file` - The data file to get structure information from.


    - #### setStructureNumber

      ```
      public void setStructureNumber(int number)
      ```

      Set the structure number.

      Parameters:
      :   `number` - The structure number.


    - #### switchCircled

      ```
      public void switchCircled()
      ```

      Switch circled nucleotides on or off.


    - #### writeDotBracketFile

      ```
      public void writeDotBracketFile(String outFile)
      ```

      Write a dot bracket file of all structures on this dialog.

      Parameters:
      :   `outFile` - The file to write.


    - #### writeHelixFile

      ```
      public void writeHelixFile(String outFile)
      ```

      Write a helix file from the current structure on this dialog.

      Parameters:
      :   `outFile` - The file to write.


    - #### writePostscriptFile

      ```
      public void writePostscriptFile(String outFile)
      ```

      Write a Postscript file from the current structure on this dialog.

      Parameters:
      :   `outFile` - The file to write.


    - #### writeSVGFile

      ```
      public void writeSVGFile(String outFile)
      ```

      Write an SVG file from the current structure on this dialog.

      Parameters:
      :   `outFile` - The file to write.


- Overview
- Package
- Class
- Tree
- Index
- Help

- Prev Class
- Next Class

- Frames
- No Frames

- All Classes

- Summary:
- Nested |
- Field |
- Constr |
- Method

- Detail:
- Field |
- Constr |
- Method
